# Functional evolutionary convergence of long noncoding RNAs involved in embryonic development

**DOI:** 10.1038/s42003-023-05278-z

**Published:** 2023-09-05

**Authors:** Ane Olazagoitia-Garmendia, Rodrigo Senovilla-Ganzo, Fernando García-Moreno, Ainara Castellanos-Rubio

**Affiliations:** 1 University of the Basque Country, UPV-EHU, Leioa, Spain; 2 Biobizkaia Health Research Institute, Barakaldo, Spain; 3 Achucarro Basque Center for Neuroscience, Leioa, Spain; 4 Ikerbasque, Basque Foundation for Science, Bilbao, Spain; 5 CIBERDEM/CIBERER, Madrid, Spain

**Keywords:** Long non-coding RNAs, Molecular evolution

## Abstract

Long noncoding RNAs have been identified in most vertebrates, but the functional characterization of these molecules is challenging, mainly due to the lack of linear sequence homology between species. In this work, we aimed to find functional evolutionary convergent lncRNAs involved in development by screening of k-mer content (nonlinear similarity) and secondary structure-based approaches combining in silico, in vitro and in vivo validation analysis. From the Madagascar gecko genes, we have found a non-orthologous lncRNA with a similar k-mer content and structurally concordant with the human lncRNA *EVX1AS*. Analysis of function-related characteristics together with locus-specific targeting of human *EVX1AS* and gecko *EVX1AS-like* (i.e., CRISPR Display) in human neuroepithelial cells and chicken mesencephalon have confirmed that gecko *EVX1AS-like* lncRNA mimics human *EVX1AS* function and induces *EVX1* expression independently of the target species. Our data shows functional convergence of non-homologous lncRNAs and presents a useful approach for the definition and manipulation of lncRNA function within different model organisms.

## Abstract (web summary)

A strategy to discover equivalent lncRNAs by k-mer content and secondary structure similarity, reveals functional convergence of embryonic development related lncRNA EVX1AS.

## Introduction

Long noncoding RNAs (lncRNAs) are RNA molecules longer than 200 bp in length that do not have coding potential^1^. LncRNAs are gaining importance due to their involvement in a wide range of biological processes, and some of them have been described to be implicated in different aspects of embryonic development^2^. However, the study of lncRNA relevance through their evolutionary conservation has been challenging due to their lack of linear sequence homology among species^3–5^. Evolutionary conservation is widely used as an indicator of the functional significance of newly discovered genes, and the simple search for homology at the nucleotide level has proven to be valuable for protein-coding genes. However, lncRNAs with similar functions often lack linear sequence homology which implies lncRNA function cannot be readily assigned from their nucleotide sequence. K-mer-based comparison methods have been demonstrated to be useful to find functionally related lncRNAs with different spatial arrangements of related sequence motifs, where a k-mer is defined as all possible combinations of a continuous sequence of nucleotides of a given length k. K-mer-based classification has been demonstrated to be a powerful approach to detect recurrent relationships between motif sequence and function in lncRNAs even in the lack of evolutionary conservation^6,7^. In addition, lncRNAs with the same secondary or tertiary structure can exert identical molecular functions despite divergent nucleotide sequences^8^, thus analyzing structural equivalence could also help identify lncRNAs with evolutionary preserved mechanisms^9^. Moreover, the molecular role of lncRNAs is tied to other characteristics such as the subcellular localization, the abundance within the cell or the interactions with other molecules^10,11^.

In this work, we have taken advantage of k-mer and structure similarity analyses to find evolutionary convergent lncRNAs involved in development. For this purpose, we used Madagascar ground gecko (*Paroedura pictus*) as the target species to find functionally conserved lncRNAs related to embryonic development. The Madagascar gecko is a useful species to investigate the evolutionary path of vertebrate features due to its phylogenetic position within the *squamates* order of reptiles^12,13^. In addition, we have performed several in vitro and in vivo analyses to investigate the functional cross-species conservation of the candidate lncRNAs. For the in vivo analyses, we have used chicken (*Gallus gallus*) embryos as an extra-phyletic species to both human and gecko species. The chicken is the most suitable animal model for experimental embryology, and it is a perfect playground for genetic engineering during development^14–16^. Using these approaches, we found that human *EVX1AS* and gecko *EVX1AS*-like regulate the coding gene *EVX1* independently of the recipient species molecular machinery. This evolutionary functional convergence of the two non-syntenic lncRNAs emerged independently in the two species, evolving in parallel to play equivalent functions from non-orthologous sequences.

## Results

### Nonlinear sequence similarity between human and gecko embryogenesis lncRNAs

**Fig. 1:**
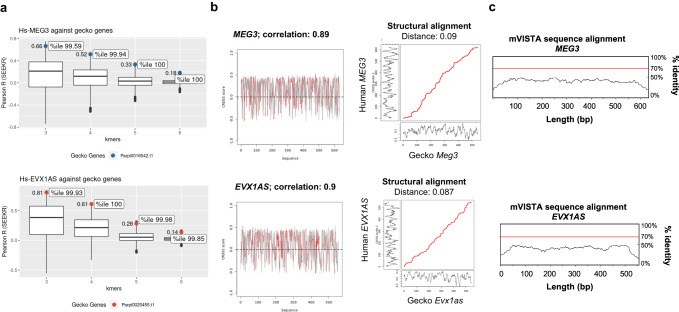
Nonlinear sequence similarity between human and gecko embryogenesis lncRNAs. **a** *k* = 3 to *k* = 6 k-mer content analysis of *MEG3* and *EVX1AS* lncRNAs using SEEKR tool. Candidate lncRNAs were compared against the Madagascar gecko genome. The boxplot represents all gecko genes, and the dots highlight the position of the selected candidate genes as well as its correlation values (Pearson, SEEKR) and its percentile (%ile). Blue and red dots correspond to the *MEG3* and *EVX1AS* genes respectively. **b** Secondary structure similarity study using CROSSALIGN tool. Optimal matching region by OBE-DTW algorithm (left) and overall structural similarity by Standard-DTW (right). Structural profiles are obtained with CROSS Global Score for the two RNAs (score >0 means a double-stranded nucleotide; <0 single-stranded). **c** Linear conservation analysis of *MEG3* (top) and *EVX1AS* (bottom) performed using mVISTA. Human sequence is shown on the *x* axis and percentage similarity to the corresponding gecko sequence on the *y* axis. The graphical plot is based on sliding-window analysis of the underlying genomic alignment. A 100-bp sliding window is at 25-bp nucleotide increment is used.

We hypothesized that functionally related lncRNAs involved in embryonic development could harbor related motif contents, although lacking linear sequence similarity. To test this, we used the SEEKR (sequence evaluation from k-mer representation) standalone^6^ to find development-associated lncRNAs (with already described functions in humans) that could be functionally convergent between human and gecko. Using the All human lncRNA (Gencode v41) set as a normalization set, we calculated the k-mer profile from *k* = 3 to *k* = 6 k-mer lengths of human *NEAT1, MEG3*, and *EVX1AS* against all sequences from *P. picta* genome v1 in order to find equivalent lncRNAs^17^. For *MEG3* and *EVX1AS*, candidate noncoding transcripts were selected based on their noncoding nature, according to the Coding Potential Calculator^18^, and its high score in all k-mer analyses (*Pp-MEG3-like* and *Pp-EVX1AS-like* from now on). Both candidates were present in the highest percentiles (>99 percentile) in all k-3 to k-6 k-mer analyses (Fig. 1a). However, no such candidate was found for *NEAT1*. To verify the validity of this approach, we also compared the k-mer content distribution between human and mouse lncRNAs as they are known to be functionally conserved. These comparisons resulted in comparable percentile values to those found between human and gecko lncRNAs with 100 percentile for *MEG3* and >97 percentile for *EVX1AS*. To evaluate the specificity of our results, we also performed the reverse analysis and compared our gecko candidates with the human set of all human lncRNAs. The results of this reverse SEEKR analysis, showed that our lncRNA candidates maintain a high k-mer correlation and that they are in the highest percentile (>96) in all k-mer analyses (from *k* = 3 to *k* = 6) (Supplementary Fig. 1a). We also confirmed the balanced GC-content of our transcripts^19^ (58% and 67% for human *MEG3* and gecko *MEG3*-like, 57% and 52% for human *EVX1AS* and gecko *EVX1AS*-like). All together, these results suggested these selected gecko noncoding RNAs are the most likely functional equivalents to human lncRNAs and ruled out the possibility of the candidates having been selected by chance.

Given that the gecko noncoding transcripts were 6-to-8 times longer than their human counterparts (4448 bp vs 554 bp for *EVX1AS* and 3751 bp vs 632 bp for *MEG3)*, we used CROSSalign^9^ to identify regions of structural similarity between the different length profiles (Fig. 1b). Both lncRNA pairs presented a structural distance lower than 0.095 (where 0 means identical structural profiles) and a 90% structure correlation. The normalized structural distance between the secondary structure profiles of *EVX1AS* was calculated as 0.087 (*P* value = 0.001) with a correlation of 90%. In the case of *MEG3*, the distance was 0.09 (*P* value = 0.01) with a correlation of 89%. Dinucleotide shuffled sequences of the gecko lncRNAs showed structural distances higher than 0.09 in both cases (0.097 for *EVX1AS* and 0.092 for *MEG3*).

Interestingly, we did not find any specific regions of conventional linear sequence homology between the pairs of structurally equivalent lncRNAs by mVISTA using the 70% conservation over a 100-bp window criteria, neither by a less stringent DotPlot analysis (with a 20% similarity) (Fig. 1c and Supplementary Fig. 1b)^20,21^.

Thus, our analysis revealed the existence of a pair of lncRNAs with high levels of k-mer similarity and significant structural equivalency between two distant vertebrate species. Together, it suggested that these lncRNAs could play similar functions in embryonic development despite a lack of linear sequence similarity.

### Human *EVX1AS* and gecko *EVX1AS-like* are principally expressed in the brain

**Fig. 2:**
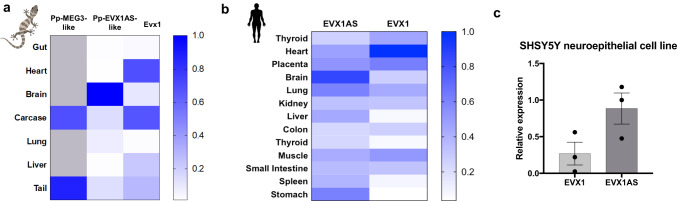
Human *EVX1AS* and gecko *EVX1AS-like* are principally expressed in the brain. Heat map showing relative expression (from 0 to 1) of (**a**) gecko *MEG3-like, EVX1AS-like* and *Evx1* and **b** human *EVX1AS* and *EVX1* in a range of tissues. Human and gecko *RPLP0* were used as housekeeping control and normalizations were done to the highest value in each experiment. Elements on the image were added using Biorender. **c** *EVX1AS* and *EVX1* expression analysis in the human neuroepithelial cell line SHSY5Y. Human *RPLP0* was used as housekeeping control. Data represents the media and standard error of three independent analyses.

In order to verify if the gecko lncRNA candidates were actually expressed, we quantified the expression of the *Pp-MEG3-like* and *Pp-EVX1AS-like* in different tissues from pre-hatching geckos. *Pp-MEG3-like* was only expressed in the tail and the carcase (Fig. 2a), with a very similar expression level in the two tissues. Expression analysis of *Pp-EVX1AS-like* demonstrated that this lncRNA is widely and tissue-specifically expressed in this species, showing the highest expression in the brain, 9–280 times higher than in the rest of the tissues (Fig. 2a and Supplementary Fig. 2a). LncRNA *Evx1as* has been described to transcriptionally regulate its nearby coding gene *Evx1*^22^. Analysis of gecko *Evx1* expression in the embryonic tissues showed the wide characteristic expression of this coding gene, with the highest expression levels in the heart and the carcase and the lowest in the lung (Fig. 2a and Supplementary Fig. 2a). We also analyzed the expression of both *EVX1AS* and *EVX1* in an RNA pool of different human tissues purchased from Clontech. As previously observed in gecko embryos, *EVX1AS* was widely expressed among the different tissues with the highest expression also present in the brain (with 1.5–12 times higher values) and the lowest in the colon and the thymus (Fig. 2b and Supplementary Fig. 2b). Regarding the coding gene *EVX1*, it likewise presented a broad tissue expression with the highest levels in the kidney and the lowest also in the lung (Fig. 2b and Supplementary Fig. 2b). Given that human brain tissue is not an accessible material, we also evaluated the expression of *EVX1AS* and *EVX1* in the human neuroepithelial cell line SHSY5Y. Both genes were expressed in this cell line confirming the validity of this culture model for further *EVX1AS* analysis (Fig. 2c).

Given the multi-level similarities, from tissue expression and structure to motif content, we selected *Pp-EVX1AS-like* lncRNA for further analysis of its potentially shared function.

### Human *EVX1AS* and gecko *EVX1AS*-*like* are likely to have evolved from independent ancestor sequences

In contrast to coding genes, it has been described that there are ortholog lncRNAs with limited linear sequence similarity due to molecular divergence through evolution. However, these ortholog lncRNAs share common synteny and some *k*-mers or regions are conserved to retain its functionality^23^. Hence, despite no significant linear sequence similarities could be detected between human *EVX1AS* and gecko *EVX1AS-like* (Fig. 1c and Supplementary Fig. 1b), we further evaluated the potential evolutionary relationship of our gecko lncRNA.

To this aim, we first used UCSC genome browser to visualize the conservation in the vicinity of the *EVX1* gene and to analyze the evolutionary origin and dynamics of *EVX1AS* transcript. As observed in Supplementary Fig. 3a, while sequence conservation of the *EVX1* gene is conserved in the different species, *EVX1AS* alignment starts decreasing in the mouse and is totally lost from the lizard on.

**Fig. 3:**
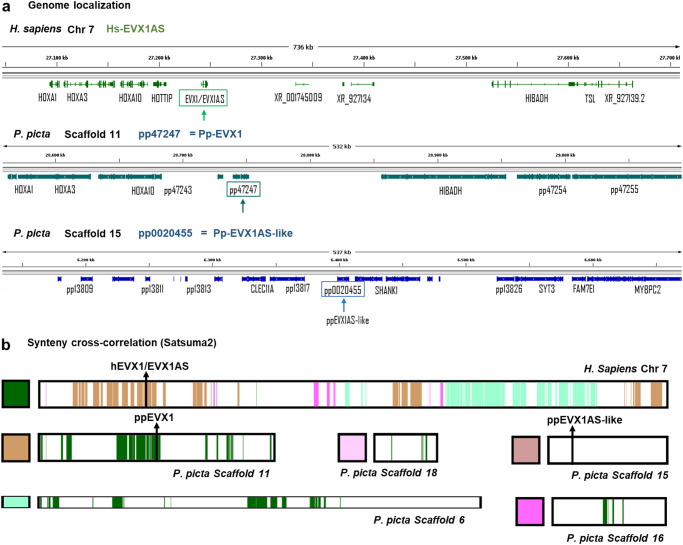
Human *EVX1AS* and gecko *EVX1AS-like* are likely to have evolved from independent ancestor sequences. **a** Genome localization of human *EVX1AS* and gecko *EVX1AS-like* genes. Top: Genomic locus for *EVX1AS* human gene (green). Bottom: Locus of its functionally equivalent gecko’s gene, pp0020455 ≈ *pp-EVX1AS-like* (blue). Genome visualization is carried out by Integrative Genome Viewer (IGV) with Human (GRCh38/hg38, RefSeq.gtf) and *P. picta* (v2, BRAKER.gtf) genomes, respectively. Except for pp0020455, which retains the V1 annotation. **b** Synteny cross-correlation (Satsuma2). Human chromosome 7 sequence alignment against gecko chromosome scaffolds. Only *EVX1AS-like* (no match) and scaffolds with match alignments have been shown for simplicity. The color code represents the chromosome aligning in the colored regions (e.g., all gecko genes display dark green colored regions due to its cross-alignment with human chromosome 7, dark green). *EVX1AS-*related genes are indicated with a bar where they are located in the chromosome.

Moreover, we also compared the colocalization of genetic loci (“synteny”) between human and gecko. Initial analysis of the genomic neighborhood (Fig. 3a and Supplementary Fig. 3b) of both lncRNAs in each species revealed great differences in the synteny of both transcripts. However, to further validate the appearing independent origin of our lncRNA, we employed Satsuma2 and GENESPACE pipeline^24^, which includes orthofinder^25^ and MCScanX^26^. As *P. picta* proteome is based on genome-annotation translation (*Transdecoder*, Haas, BJ https://github.com/TransDecoder/TransDecoder), Satsuma2^27^ allowed us to compare large and complex DNA sequences (whole-genome comparison). Synteny results showed no cross-correlation between human *EVX1AS* chromosome 7 and gecko *EVX1AS*-like genomic loci (Fig. 3b).

Nonetheless, we carried out GENESPACE pipeline to assess the existence of orthologous gecko *EVX1* orthogroup loci in gecko. Both orthofinder and MCScanX define *pp47247 as* the orthologous of human *EVX1*, which is in scaffold 11. This is a distinct chromosome from *pp-EVX1AS-like* and does present cross-correlation with the *EVX1* region in human chromosome 7 by both Satsuma2 and MCScanX (Fig. 3b). Among the derived transcripts of *pp47247*, we have not detected any long noncoding transcript (CPC2) that could reproduce *EVX1/EVX1AS* model. However, it would be interesting to obtain further transcriptomic evidence by deep noncoding RNA sequencing.

As *Paroedura picta’s* genome is not based on long read data, we wanted to further confirm the genomic sequence of *EVX1AS* as well as its synteny. We amplified and re-sequenced our gecko *EVX1AS-like* candidate and confirmed that the gecko locus corresponding to our candidate lncRNA is correctly assembled in the scaffold 15. Thus, gecko *EVX1AS-like* localizes in scaffold 15, while gecko *Evx1* ortholog is in scaffold 11.

To sum up, all our synteny analyses indicate that both human *EVX1AS* and gecko *EVX1AS-like* evolved separately, from independent ancestor sequences, but converged into similar characteristics in parallel. Thus, we delved into these analog function-related characteristics.

### Human *EVX1AS* and gecko *EVX1AS-like* share function-related characteristics

**Fig. 4:**
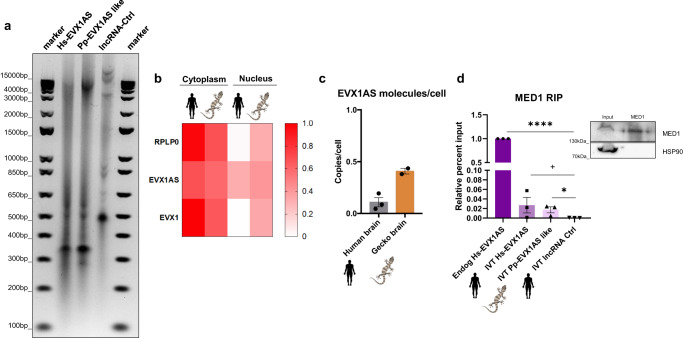
Human EVX1AS and gecko EVX1AS-like share function-related characteristics. **a** Mobility assay of in vitro transcribed (IVT) human (Hs) and gecko (Pp) *EVX1AS-like*. A non-related lncRNA was used as a control (Ctrl). **b** Cellular localization of human *EVX1AS* and gecko *EVX1AS-like* in human SHSY5Y cells (*n* = 3) and gecko brains (*n* = 2). *PO* and *EVX1* were used as cytoplasmic controls. **c** Analysis of *EVX1AS* and *EVX1AS*-like copy numbers in human and gecko brains, respectively. Data represents the mean and standard error of the experiments. **d** Analysis of endogenous and in vitro transcribed *EVX1AS* variants levels bound to MED1 protein after immunoprecipitation. An in vitro transcribed unrelated lncRNA was used as a control. (*n* = 3) A representative western blot of the immunoprecipitation is shown. (+*P* < 0.1, *P < 0.05, ****P < 0.0001 according to one-tailed Student’s *t* test). Elements on the image were added using Biorender.

RNA structure, subcellular localization, and abundance of lncRNAs are generally related to their function and molecular roles. Thus, to experimentally assess their functional convergence, we evaluated several function-related characteristics (structure, localization and abundance) for both human and gecko *EVX1AS possible analogues*. These two lncRNA forms showed an almost identical migration pattern when in vitro transcribed lncRNAs corresponding to the structural analog region were migrated in a nondenaturing agarose gel (Fig. 4a). Conversely, an unrelated in vitro transcribed lncRNA, which was used as a control, showed a totally different migration pattern, confirming the equivalency of the secondary structures of human and gecko *EVX1AS* analogues predicted in silico (Fig. 1b).

For subcellular localization assessment, the amounts of *EVX1AS* were quantified in whole and nuclear fractions of human neuroepithelial cells and the gecko brain. As previously described^22^, *EVX1AS* was found in both cellular compartments suggesting that apart from regulating *EVX1* in cis, this lncRNA also exerts other yet undescribed biological functions. The percentage of *EVX1AS* present in the nucleus was very similar in both species (approximately 30%) (Fig. 4b), further supporting its functional convergence. Evaluation of *EVX1AS* abundance was performed using human and gecko brain cDNA and a reference plasmid of each lncRNA (Supplementary Fig. 4a). We had the limitation that while the gecko brains tested were embryonic, human brain samples were from adult individuals. However, the amount of *EVX1AS* molecules per cell in both cases was lower than 1 (Fig. 4c), pointing to a cell type-specific expression of the lncRNA within the brain. The higher amount of copies per cell present in the gecko brain may be suggestive of a high peak of activity of this lncRNA regulating *EVX1* during embryonic development.

The regulation of mouse *Evx1* expression by *Evx1as* has been described to be mediated by the binding of the lncRNA to the mediator complex^22^. The mediator complex is a multiprotein complex that functions as a transcriptional coactivator and interacts with a wide range of proteins. MEME motif analysis of our lncRNAs revealed that despite differentially located within their sequence (even in opposite sense) there are several common enriched protein binding motifs within both lncRNAs (Supplementary Fig. 4b). We also used lncLOOMv2^28^, which searches for synteny conserved *k*-mers. However, none of the ordered 6-9 nucleotide motifs in both lncRNA sequences (Supplementary Fig. 4c) matched with a TargetScan functional motif, supporting our previous synteny results.

Interestingly, alignment of the MEME-enriched motifs using TOMTOM Motif comparison tool showed enrichment of several brain function-related protein binding motifs, as MATR3, HNRNPA1 and PTBP1 (Supplementary Fig. 4d), that have been reported to interact with members of the mediator complex according to GENEMANIA server^29^. Thus, to analyze if both our lncRNA forms also bind the mediator complex, we performed an RNA immunoprecipitation experiment of MED1 protein using lysates from SHSY5Y cells. Immunoprecipitation of human MED1 was able to retrieve both, endogenous and IVT human *EVX1AS*. In addition, we also observed the lncRNA-MED1 interaction when IVT *Pp-EVX1AS-like* was added to the protein lysates. Conversely, our IVT lncRNA control did not interact with MED1, confirming the specificity of the binding (Fig. 4d).

Equivalence in structure, subcellular localization and abundance of *EVX1AS* from human and *EVX1AS-like* from gecko, together with the interaction with the mediator complex, strongly suggested a shared cellular function.

### Human *EVX1AS* and gecko *EVX1AS-like* are functionally convergent

**Fig. 5:**
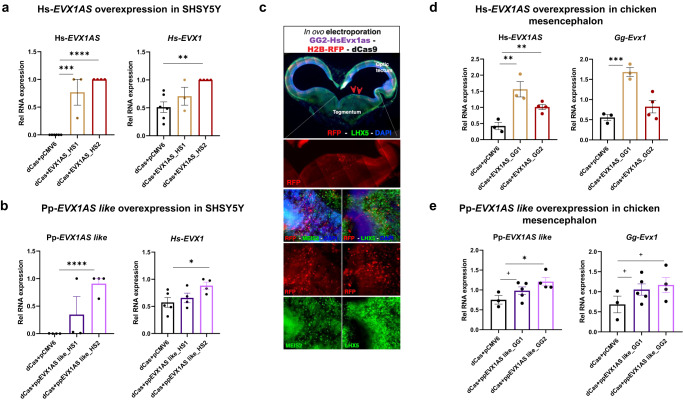
*EVX1AS* analogues are functionally convergent among species. **a** Relative RNA expression of human *EVX1AS* lncRNA and *EVX1* upon human *EVX1AS* overexpression in SHSY5Y cells. **b** Relative RNA expression of gecko *EVX1AS-like* lncRNA and human *EVX1* upon pp-*EVX1AS*-like overexpression in SHSY5Y cells. Human *RPLP0* was used as housekeeping control and normalizations were done to the highest value in each experiment. **c** Immunohistochemistry of the chicken embryonic midbrain on coronal section showing the successful transfection of the lncRNA plasmid. Transfected cells are shown in red due to their ectopic expression of red fluorescent protein RFP. LHX5 and MEIS2 patterns of expression (in green) indicate the mesencephalic region that was actually transfected. DAPI counterstain in blue. **d** Relative RNA expression of human *EVX1AS* lncRNA and chicken *Evx1* upon human *EVX1AS* overexpression in chicken mesencephalon. Scale bars represent 1 mm, 500 micras and 100 micras from top to bottom. **e** Relative RNA expression of gecko *EVX1AS-like* lncRNA and chicken *Evx1* upon *pp-EVX1AS-like* overexpression in chicken mesencephalon. Chicken *Rplp7* was used as housekeeping control. Data represents the mean and standard error of 3–6 independent experiments. (+*P* < 0.1, *P < 0.05, **P < 0.01, ***P < 0.001, ****P < 0.0001 according to one-tailed Student’s *t* test). HS1 and HS2: sgRNAs targeting human *EVX1* TSS1 and TSS2, respectively. GG1 and GG2: sgRNAs targeting chicken *Evx1* TSS1 and TSS2, respectively.

To analyze whether the previous results were representative of a functional in-cell equivalence, we conducted overexpression experiments of both lncRNAs in two experimental paradigms: in vitro, in SHSY5Y cells; and in vivo, in chick embryonic brains. For this approach, we used the CRISPR-Display technique^30^, in order to specifically localize *EVX1AS* into the promoter of human or chicken *EVX1*. For in vitro experiments, we cloned either the human or gecko *EVX1AS* lncRNA preceded by two different guide RNAs specific for human *EVX1* promoter (HS1 and HS2 being sgRNAs targeting human *EVX1* TSS1 and TSS2, respectively). Otherwise, for in vivo experiments, gRNAs for chicken *Evx1* promoter were designed (GG1 and GG2 as sgRNAs targeting chicken *Evx1* TSS1 and TSS2 respectively). These constructs were followed by a 3’ box and cloned into a CMV-driven vector to guide and tether the *EVX1AS* RNA to the *EVX1* promoter (Supplementary Fig. 5a, b). The transfection of Hs-*EVX1AS* vectors into the SHSY5Y cells showed a 4-13 × 10^6^ fold statistically significant overexpression (*P* < 0.01 for HS1 and *P* < 0.001 for HS2) (Fig. 5a). Using Pp-*EVX1AS-like* plasmids, 1.5–3 × 10^6^ fold overexpression could be achieved, being significant only with HS2 gRNA (*P* < 0.001) (Fig. 5b). The directed overexpression of the human *EVX1AS* was able to induce the expression of *EVX1* mRNA in the SHSY5Y human neuroblastoma cell line about two times (*P* < 0.01 for HS2) (Fig. 5a). When gecko *EVX1AS-like* was overexpressed in the human cells, we also observed a 1.5–2-fold increase in the mRNA levels of *EVX1* (*P* < 0.01 for HS2) (Fig. 5b), similar to what it was previously observed using this same approach^22^. No induction of *GAPDH* negative control could be observed in either of the cases (Supplementary Fig. 5c, d) confirming that these two lncRNAs functionally converged in their role of controlling *EVX1* mRNA expression. Interestingly, the tethering mediated by the sgRNA2 (named HS2) (closer to the *EVX1* start codon) performed more efficiently, independently of the origin of the lncRNA. To further confirm the functional convergence of human and gecko *EVX1AS analogues*, we tethered an unrelated human lncRNA to *EVX1* promoter using the most efficient sgRNA (HS2). We confirmed that tethering of this lncRNA does not induce the expression of *EVX1* (Supplementary Fig. 5e, f), supporting that only *EVX1AS* homologs lead to upregulation of *EVX1*.

To further confirm these data, we decided to use an extra-phyletic in vivo model that could act as a recipient for both species. The chicken embryo is a good model for these analyses as it represents a suitable experimental model that is phylogenetically distant from both human and gecko. As it has been previously described, we found expression of the *Evx1* coding gene in the chicken embryo mesencephalon, but not in the forebrain (Supplementary Fig. 5g). We electroporated our lncRNAs together with the catalytically dead Cas9 into the mesencephalon of embryonic day 3 (E3) chicken embryos, when the mesencephalic *Evx1* expressing cells are mostly being generated^31,32^. We validated our strategy using immunofluorescence, as we confirmed that we were able to electroporate and express our plasmids in the mesencephalic region of chicken embryos as determined by LHX5 and MEIS2 expression (Fig. 5c and Supplementary Fig. 5h). Three days after overexpression (E6), as previously observed in the human cells, the tethering of both human and gecko *EVX1AS* analogues to the chicken *Evx1* promoter induced the levels of *Evx1* mRNA between 1.2- and 3.5-fold (*P* < 0.001 for *Hs-EVX1AS* and *P* < 0.08 for *Pp-EVX1AS-like*) (Fig. 5d, e). *Gapdh* expression did not vary significantly (Supplementary Fig. 5i, j); confirming the functional convergence of the human and gecko lncRNAs.

The ability of the human and gecko *EVX1AS* analogues to regulate the expression of human and chicken coding orthologous gene *EVX1* in all the combinations (human–human, gecko–human, human–chicken, gecko–chicken) confirms the functional similarity of both lncRNAs, and points to a case of evolutionary convergence.

## Discussion

LncRNAs regulate fundamental cellular processes, such as embryonic development, but the physiological importance of the majority of lncRNAs remains to be identified^33^. The lack of strategies to find relationships between lncRNA sequence and mechanism makes it difficult to functionally classify them. In addition, the mechanistic studies using human material have also great limitations, making the functional characterization of lncRNAs a very difficult task^34,35^. Moreover, the absence of linear conservation for these transcripts makes the identification of ortholog or functionally equivalent lncRNAs among different species very challenging^36^, especially between evolutionarily distant species such as gecko and human. Thus, the need to develop a strategy to investigate functionally equivalent lncRNAs, bearing in mind that lack of sequence conservation does not directly imply different functions, will be of great importance to fully understand the evolution and function of these emerging regulatory elements^8^.

In this study, we have used SEEKR algorithm for nonlinear comparison of lncRNAs to identify potential gecko functional counterparts of human lncRNAs involved in development. After this initial screening, the selected candidates were evaluated based on their noncoding potential and their structural similarity with CPC2 and CROSSAlign, respectively. These in silico analysis led us to two candidate lncRNAs that could perform the same functions as human lncRNAs *MEG3* and *EVX1AS*.

*MEG3* has been involved in pluripotency and reprogramming and it is mainly expressed in the hypophysis^37,38^. The expression analysis of *Pp-MEG3-like* in the gecko tissues only showed expression in the tail and the carcase suggesting a lack of functional equivalence with the human lncRNA. On the other hand, *EVX1AS* has been related with the mesoderm differentiation and it is known to regulate the expression of *EVX1* coding gene, which is expressed in the midbrain during embryonic development^22,39^. We found that *Pp-EVX1AS-like* was mainly expressed in the embryonic gecko brain, being a suitable candidate for further function-related studies.

In order to clarify if human and gecko *EVX1AS* analogues evolved independently, we further studied the genomic localization of our gecko candidate. Despite the genomic sequence plasticity of lncRNAs, short specific sequences that are mainly conserved across species have been described; pointing that there exist orthologous, functionally equivalent lncRNAs originated from a common ancestral gene. Indeed, many authors have relied on synteny or genomic localization to discover orthologue counterparts to human lncRNAs^5,23,40,41^. However, this approach leaves aside the role of lncRNAs as a source of evolutionary innovation, not considering the possibility of functionally convergent evolution of lncRNAs.

According to our results, human *EVX1AS* and gecko *EVX1AS-like* are highly unlikely to be orthologous despite their high functional similarity. The syntenic analysis indicated no linear sequence similarity between the gecko *EVX1AS-like* scaffold 15 and the human *EVX1AS* chromosome 7. On the contrary, there exists an orthologous coding *EVX1* in the gecko scaffold 11 that shares an equivalent genomic locus to human *EVX1/EVX1AS*. Thus, an event of translocation of *EVX1AS* to the scaffold 15 with no conservation of its genomic vicinity nor its overlapping coding *EVX1* seems highly unlikely. In addition, evaluation of conservation of *EVX1/EVX1AS* locus across species showed that while *EVX1* coding gene is conserved among different species, *EVX1AS* conservation is already reduced in mouse and it is totally lost from lizard on. This observation is in accordance with previous works and supports the idea that most lncRNAs only have short functional motifs in common^40^. Furthermore, we have proved the existence of functional motifs shared by both lncRNAs. Nonetheless, the sequential order of these equivalent functional motifs within the molecule is quite different. Contrarily, when the order of motifs is fixed, as for lncLOOMv2^28^, no functional syntenic motif shared by both lncRNAs could be detected. This is another indicator of the absence of a common genomic ancestor between human *EVX1AS* and gecko *EVX1AS-like* lncRNAs, which are highly likely to have evolved independently, in parallel, so that their structures and functions converged in both human and gecko.

As previously stated, lncRNAs acquire complex structures that usually dictate their function. Moreover, lncRNAs can be found throughout the cell, acting in a wide range of cellular processes, and are commonly lowly expressed^11,42^. These characteristics—structure, subcellular location, level of expression—would be common to those lncRNAs that exert the same functions, helping in the evaluation of putative convergent lncRNAs. The identical gel migration pattern of *Pp-EVX1AS-like* and *Hs-EVX1AS*, together with the ubiquitous localization and the scarce expression of both lncRNAs suggested that they could be playing parallel functions. Moreover, these lncRNAs presented common motifs known to recruit RNA-binding proteins, providing additional insight into similarities between the two lncRNAs. Interestingly, the three RNA-binding proteins predicted to bind the human and gecko lncRNAs showed interactions with different members of the mediator complex, as reported by the GENEMANIA server^29^. Consistent with these data, we were able to show that human *EVX1AS* and gecko *EVX1AS-like* physically interact with MED1 protein, which has been described to bind *EVX1AS* for *EVX1* transcription regulation^22^, further supporting their functional similarity.

*EVX1AS* is known to be involved in regulatory processes within the nucleus, i.e., regulating the expression of its nearby coding gene *EVX1*^22^. Although the mechanistic dissection of these types of regulators in vivo is technically challenging, we took advantage of the CRISPR-Display technique, which is a very useful tool for relocating lncRNA transcripts to an ectopic site^30^. This technique allowed us to direct human *EVX1AS* and gecko *EVX1AS-like* to the promoter of human and chicken *EVX1* coding gene, regardless of the lncRNA and recipient species. Using this approach, we showed that both human and gecko *lncRNAs* are able to regulate *EVX1* expression in both model systems, human and chicken, a significant proof of functional equivalence.

Overall, our study provides an innovative strategy to discover equivalent lncRNAs by *k*-mer content and secondary structure similarity, but also to validate their functional resemblance based on their expression, localization, abundance, in vitro and in vivo molecular studies. To our knowledge, our approach based on nonlinear comparisons has identified for the first time a non-homologous functional convergent pair of lncRNAs and opens the door to novel research based on analogous RNAs. The existence of non-homologous lncRNAs with a shared common function and features suggests that lncRNAs have played an important role in evolutionary innovation, despite being originated by different lineages together.

## Methods

### Selection of candidate lncRNAs using *k*-mer content

Starting from previously described embryonic development-related lncRNAs, we decided to analyze *NEAT1, MEG3* and *EVX1AS* based on their length (<4 kb) and their already described conservation between mouse and human. We then analyzed the *k*-mer content of our candidate lncRNA sequences against all the sequences of our reptilian model, the Madagascar ground gecko (*Paroedura pictus*) genome v1^17^, using SEEKR 1.5.4 in Python. We used all GENCODE v41 set as a background set to derive the mean and standard deviation of the counts for each *k*-mer^6^. In addition, we did our analysis using four *k*-mer lengths: *k* = 3, *k* = 4, *k* = 5 and *k* = 6. The gecko genes with highest *k*-mer values in all *k*-mer lengths, *k* = 3 to *k* = 6, were chosen and the noncoding nature of the derived gecko transcripts was analyzed using the Coding Potential Calculator (CPC) web-based interface^18^. The reverse comparison, in which we analyzed our gecko lncRNAs of interest against all human lncRNAs and the *k*-mer content correlations between the mouse and human lncRNA forms were performed as controls. All boxplots from SEEKR results displayed in the results and supplementary figures were created in *R*, using *ggplot2* package and SEEKR csv results.

### RNA secondary structure

The in silico structural equivalence of the gecko and the human sequences was analyzed using CROSSalign^9^. First, the shorter profile of each couple was searched in the bigger one using the OBE-DTW procedure. Secondly, we performed Standard-DTW analysis to assess the structural alignment of the equivalent regions. The main output of this analysis is the structural distance, between the two input structures. The closer the distance is to 0 (with 0 meaning identical structural profiles) the higher the similarity in term of secondary structure. RNA molecules with a structural distance of 0.095-0.10 or higher are to be considered different in terms of secondary structure. For control comparisons in CROSSalign we generated random lncRNA sequences of the same dinucleotide frequency (even the same number of each dinucleotide) as that of gecko RNAs using the web server of the Clote computational biology lab (http://clavius.bc.edu/~clotelab/RNAdinucleotideShuffle/). These random sequences were used to perform the Standard-DTW structural distance analysis with the human lncRNAs.

A Jupyter Notebook has been uploaded on GitHub to follow all the steps for *k*-mer and CROSSalign analysis in a Python3 environment: https://github.com/rodrisenovilla/Olazagoitia-Garmendia/blob/ffbeb12b889791b4b0c24f47cff4436cce2979eb/seekr.ipynb.

For mobility assessment, the structural homolog regions from human and gecko *EVX1AS* were in vitro transcribed (IVT) using a T7 RNA Polymerase (Takara Bio, San Jose, CA). IVT lncRNAs were then purified, heated at 95 °C for 3 min, placed on iced and run in a nondenaturing agarose gel in TBE.

Human lncRNA *lncTGM2* has been used as a negative control, a lncRNA that is also expressed in neurons but has an *EVX1-*unrelated function^43^. *LncTGM2* does not present a high *k*-mer similarity (k-3:0.28, k-4:0.19) or equivalent secondary structure (structural distance to human *EVX1AS* 0.103 and to gecko *EVX1AS*-like 0.1) based on SEEKR and Crossalign analysis respectively.

### Linear sequence similarity analysis

Dot plots were generated using EMBOSS dotmatcher^21^. We used a window size of 5 and a threshold of 25.

mVISTA was used to visualize linear sequence alignments^20^. We used a 100-bp sliding window with a 25-bp nucleotide increment for the analysis of the underlying genomic alignment. The similarity was set at 70%.

### Synteny analysis (genomic loci analysis)

lncLOOMv2 analyses^28^ were carried out as detailed in its GitHub (https://github.com/lncLOOM/LncLOOMv2) with default parameters.

For further synteny analyses, although current genome version annotation^44^ is under construction, we have explored the synteny of the analog lncRNAs of interest in this chromosome-scale genome version. Based on the v1 version, the gecko gene of interest was located by BLASTn^45,46^ in the v2. We visualized the closest genes by Integrative Genome Viewer (IGV, https://igv.org/)^47^ and we retrieved the functional annotation of the closest genes by EggNOG (https://github.com/eggnogdb/eggnog-mapper)^48^. When no gene homolog was identified regarding the EggNOG standards, the default gene name for the new annotation was kept (e.g., *pp13819*).

Satsuma2 (https://github.com/bioinfologics/satsuma2)^27^ was employed to cross-align gecko genome against human chromosome 7 (*Ensembl* GRCh38 release 109), where human *EVX1AS* is located. The default parameters were used for SatsumaSynteny2 (cross-aligment), BlockDisplaySatsuma and ChromosomePaint. The pipeline followed to obtain the output plot displayed is available on our github. To cross-check Satsuma2 results, GENESPACE v1.1.5 (https://github.com/jtlovell/GENESPACE)^24^ pipeline was carried out to assess the existence of orthologous based on orthofinder (https://github.com/davidemms/OrthoFinder)^25^ and MCScanX: Multiple Colinearity Scan Tool (https://github.com/wyp1125/MCScanX)^26^, which combine BLAST^45,46^ comparisons at transcript level and synteny analysis. The versions are determined and fixed by GENESPACE pipeline.

### Protein–RNA-binding motif analysis

The sequences of human and gecko *EVX1AS* were analyzed using MEME Motif Discovery tool in order to find enriched motifs within the lncRNAs. For Motif Discovery, classic mode, zoops distribution, 10 motifs, and a motif wide between 6 and 15 were selected as parameters. Additionally, discovered motifs were submitted to TOMTOM Motif comparison tool using Ray 2013 all-species RNA motif database to find proteins that would bind the enriched RNA motifs^49^.

### Subcellular localization

For quantification of *EVX1AS* levels in nuclear and cytoplasmic compartments, nuclei were isolated as described previously^50^. Briefly, cells were resuspended in C1 lysis buffer (1.28 M sucrose, 40 mM Tris-HCl pH 7.5, 20 mM MgCl_2_, 4% Triton X-100), incubated in ice for 15 min and centrifuged at 600× *g* for 15 min. The resulting pellet were isolated nuclei and RNA was extracted as usual. The amount of specific nuclear RNA measured by RT-QPCR was compared to the total amount of the RNA in the whole cell.

### Quantification of molecules per cell

In order to determine the *EVX1AS* copy number in human and gecko cells, a reference plasmid incorporating the cDNA sequence of each *EVX1AS* form was used. Absolute quantification was performed using five tenfold serial dilutions of the reference standard. Ct versus the dilution factor was plotted in a base-10 semi-logarithmic graph, fitting the data to a straight line. Plot was then used as a standard curve for extrapolating the number of molecules of *EVX1AS* in the cells.

### Gecko tissue dissection

Three pre-hatching geckos at embryonic days E50 to E55 were anesthetized by hypothermia. After the opening of the egg, the embryos were sacrificed by cervical dislocation and a collection of tissues were obtained and flash frozen in liquid nitrogen: brain, heart, eyes, gut, lungs, liver, tail, and the remaining carcase (containing mainly bones, muscle and skin).

### RNA tethering

CRISPR-display was performed as previously described^30^. Briefly, human and gecko lncRNAs fused to a U1 3’box at their 3’ end and to a scaffold at their 5’ end were ordered as gBlocks (IDT). Subsequently, sgRNAs targeting the human or chicken *EVX1* TSS (available under request) were introduced by PCR and the whole construct was cloned into a pCMV plasmid. Then, the fusion lncRNA-sgRNA constructs were co-transfected with the catalytically inactive dCas9 into SHSY5Y cells or chicken embryos (see following section). All generated plasmids are available upon request.

### Cells

The neuroepithelial SHSY5Y cell line (CRL-2266) was purchased from ATCC (Manassas, VA, USA). Cells were cultured in 50% EMEM and 50% F12 medium supplemented with 10% FBS (Millipore, Burlington, MA, USA #S0115), 100 units/ml penicillin, and 100 μg/ml streptomycin (Lonza, #17-602E). For overexpression experiments, 250 ng of each plasmid were used. In total, 150,000 cells/well were seeded and transfected using X-TremeGENE HP DNA transfection reagent (Sigma-Aldrich, #6366546001), cells were harvested after 48 h.

### Animals

All animal experiments were approved by a local ethical review committee and conducted in accordance with personal and project licenses in compliance with the current normative standards of the European Union (Directive 2010/63/EU) and the Spanish Government (Royal Decrees 1201/2005 and 53/2013, Law 32/107). Fertilized hen eggs (Gallus gallus), obtained from Granja Santa Isabel (Córdoba, Spain), were incubated at 37.5 °C in a humidified atmosphere until the required stages (Bellairs and Osmond, 2014). The day when eggs were incubated was considered embryonic day E0.

Gecko eggs were harvested from a local breeder colony of Madagascar ground geckos (*Paroedura pictus*) at Achucarro (based on the colony at the Department of Ecology of Charles University, Czech Republic). Adult geckos were maintained on a 12/12-h light/dark and temperature cycle (8 a.m. lights on; 28 °C diurnal temperature, 23 °C nocturnal temperature) and provided with ad libitum access to food and water. Eggs were incubated at 28 °C in a low-humidified atmosphere until the required stages^12^. The day when eggs were found in the terrarium was considered E0.

### In ovo electroporation

Electroporation of chick embryos was performed as previously described^51^. Briefly, eggs were incubated in a vertical position at 38 °C. Plasmids were injected with a volume of less than 1 μl into the fourth ventricle of E3 chick embryos using a fine-pulled glass needle. Four electric pulses (14–17 V, 15 ms pulses with a 950-ms interval: BTX electroporator ECM) were then applied to the brain between insulated silver 40 mm × 0.8 mm wire electrodes with flattened pole (Intracel). Drops of Ringer’s solution supplemented with antibiotics (penicillin/streptamycin: Sigma) were added to the egg. Embryos were incubated until E6, when tissue was harvested for further research.

### RNA extraction and RT-QPCR

RNA from all samples was extracted using Direct-zol RNA miniprep kit (Zymo Research, Irvine, USA, #R2053) with DNAse treatment. For the extraction of RNA from gecko and chicken tissues, samples were homogenized with a pellet pestle prior to extraction.

Overall, 500–1000 ng of RNA were used for the retrotranscription reaction using iScript cDNA Synthesis Kit (BioRad, CA, USA, #1708890). Expression values were determined by qPCR using Sybr Green (iTaq SYBR Green Supermix, BioRad, #1725124) and specific primers. *RPLP0* gene was used as endogenous control in human and in gecko and *Rplp7* in chicken samples. Reactions were run in a BioRad CFX384 and melting curves were analyzed to ensure the amplification of a single product. All qPCR measurements were performed in duplicate, and expression levels were analyzed using the 2–∆∆Ct method. To reduce the variability across experiments, we normalized the relative expression as follows: all values from the same experiment were normalized to the highest value, hence we obtained values ranging from 0 to 1. In the case of in vivo experiments, relative expression values were normalized using z-score to allow the combination of values from different qPCRs. All primer sequences are listed in Supplementary Table 1.

### RNA immunoprecipitation (RIP)

For RIP experiments, SHSY5Y cells were lysed in RIP buffer (150 mM KCl, 25 mM Tris, 0.5 mM DTT, 0.5% NP-40, PI), kept on ice for 15 min, and homogenized using a syringe. IVT lncRNAs were incubated with RNA secondary structure buffer and added to the lysates. The mixes were pre-cleared with proteinG dynabeads (ThermoFisher, Waltman, MA, USA) for 1 h in a wheel shaker at 4 °C. Pre-cleared lysates were incubated with 1 μg of MED1 antibody (Santa Cruz Biotechnologies, Dallas, TX, USA) for 1 h at room temperature. After incubation dynabeads were added and further incubated for 30 min. The immunoprecipitation was washed three times with RIP buffer, three times with low salt buffer (50 mM NaCl, 10 mM Tris-HCl, 0.1% NP-40) and three times with high salt buffer (500 mM NaCl, 10 mM Tris-HCl, 0.1% NP-40). After the washes, 70% of the beads were resuspended in RNA extraction buffer, and 30% was used for WB.

### Western blot

Laemmli buffer (62 mM Tris-HCl, 100 mM dithiothreitol (DTT), 10% glycerol, 2% SDS, 0.2 mg/ml bromophenol blue, 5% 2-mercaptoethanol) was added to the protein samples and were denatured by heat. Proteins were migrated on 8% SDS-PAGE gels. Following electrophoresis, proteins were transferred onto nitrocellulose membranes using a Transblot-Turbo Transfer System (Biorad) and blocked in 5% nonfatty milk diluted in TBST (20 mM Tris, 150 mM NaCl and 0.1% Tween 20) at room temperature for 1 h. The membranes were incubated overnight at 4 °C with primary antibodies diluted 1:500 in TBST. Immunoreactive bands were revealed using the Clarity Max ECL Substrate (BioRad, #1705062) after incubation with a horseradish peroxidase-conjugated anti-mouse (1:10,000 dilution in 2.5% nonfatty milk) secondary antibody for 1 h at room temperature. The immunoreactive bands were detected using a BioRad Molecular Imager ChemiDoc XRS and quantified using the ImageJ software (BioRad).

The following antibodies were used for western blotting: HSP90 (Cell Signaling; #4874) and MED1 (sc-74475).

### Immunohistochemistry

Embryonic chick brains were fixed by immersion in PFA (4% paraformaldehyde, PFA, diluted in phosphate-buffered saline 0.1 M– PBS, pH 7.3). Brains were transferred to PBS 6 h after fixation. Brains were sectioned in the coronal plane at 50–70-μm thickness in a vibrating microtome (Leica VT1000S). Single and double immunohistochemical reactions were performed as described previously^52^ using the following primary antibodies: rabbit antibody to histone H3-phospho S10 (Abcam ab47297; 1:1000), mouse antibody to LHX5 (DSHB;1:30) and mouse antibody to Meis2 (DSHB; 1:30). For secondary antibodies (all 1:1000), we used Alexa 647 goat antibody to rabbit IgG (Molecular Probes, A32733) and Alexa 488 goat antibody to mouse IgG (Molecular Probes, A11001). Sections were counterstained with DAPI.

### Statistics and reproducibility

The data are represented as the mean ± standard error of the mean of at least three biological replicates. Mean comparisons were performed by Student’s *t* test. The statistical significance level was set at *P* < 0.1.

### Reporting summary

Further information on research design is available in the Nature Portfolio Reporting Summary linked to this article.

### Supplementary information

Supplementary Information

Description of Additional Supplementary Files

Supplementary Data 1

Reporting Summary

## Data Availability

The files used for the bioinformatic analyses can be found in our GitHub (https://github.com/rodrisenovilla/Olazagoitia-Garmendia). The raw data from the expression studies is available as Supplementary Data.

## Code availability

Although many of the analyses were carried out in web-page apps, we have coded a pipeline to unify all our bioinformatic analysis (Olazagoitia_pipeline.ipynb), to generate the boxplots for SEEKR results (seekr_output_explore.Rmd) and to carry out GENESPACE pipeline (Genespace_pipeline.Rmd). For further explanations or find trouble re-running them, please contact the corresponding author.

## References

[1] Rinn JL, Chang HY (2012). Genome regulation by long noncoding RNAs. Annu. Rev. Biochem..

[2] Fico, A., Fiorenzano, A., Pascale, E., Patriarca, E. J. & Minchiotti, G. Long non-coding RNA in stem cell pluripotency and lineage commitment: functions and evolutionary conservation. *Cell. Mol. Life Sci.* **76**, 10.1007/s00018-018-3000-z (2019). 10.1007/s00018-018-3000-z PMC6439142 30607432

[3] Johnsson P, Lipovich L, Grandér D, Morris KV (2014). Evolutionary conservation of long noncoding RNAs; sequence, structure, function. Biochim. Biophys. Acta.

[4] Diederichs, S. The four dimensions of noncoding RNA conservation. *Trends Genet.* **30**, 10.1016/j.tig.2014.01.004 (2014). 10.1016/j.tig.2014.01.004 24613441

[5] Szcześniak MW, Kubiak MR, Wanowska E, Makalowska I (2021). Comparative genomics in the search for conserved long noncoding RNAs. Essays Biochem..

[6] Kirk JM (2018). Functional classification of long non-coding RNAs by k-mer content. Nat. Genet..

[7] Sprague D (2019). Nonlinear sequence similarity between the Xist and Rsx long noncoding RNAs suggests shared functions of tandem repeat domains. RNA.

[8] Smith MA, Gesell T, Stadler PF, Mattick JS (2013). Widespread purifying selection on RNA structure in mammals. Nucleic Acids Res..

[9] Ponti RD, Armaos A, Marti S, Tartaglia GG (2018). A method for RNA structure prediction shows evidence for structure in lncRNAs. Front. Mol. Biosci..

[10] Statello, L., Guo, C. J., Chen, L. L. & Huarte, M. Gene regulation by long non-coding RNAs and its biological functions. *Nat. Rev. Mol. Cell Biol.* **22**, 10.1038/s41580-020-00315-9 (2021). 10.1038/s41580-020-00315-9 PMC7754182 33353982

[11] Much, C. et al. Evolutionary divergence of Firre localization and expression. *RNA*10.1261/rna.079070.121 (2022). 10.1261/rna.079070.121 PMC9074896 35304421

[12] Noro M, Uejima A, Abe G, Manabe M, Tamura K (2009). Normal developmental stages of the Madagascar ground gecko Paroedura pictus with special reference to limb morphogenesis. Dev. Dyn..

[13] Nomura, T., Kawaguchi, M., Ono, K. & Murakami, Y. Reptiles: a new model for brain evo-devo research. *J. Exp. Zool. Part B Mol. Dev. Evol.* **320**, 10.1002/jez.b.22484 (2013). 10.1002/jez.b.22484 23319423

[14] Itasaki, N., Bel-Vialar, S. & Krumlauf, R. ‘Shocking’ developments in chick embryology: electroporation and in ovo gene expression. *Nat. Cell Biol.* **1**, 10.1038/70231 (1999). 10.1038/70231 10587659

[15] Nakamura, H. & Funahashi, J. Electroporation: past, present and future. *Dev. Growth Differ.* **55**, 10.1111/dgd.12012 (2013). 10.1111/dgd.12012 23157363

[16] García-Moreno F (2018). Absence of tangentially migrating glutamatergic neurons in the developing avian brain. Cell Rep..

[17] Hara Y (2018). Madagascar ground gecko genome analysis characterizes asymmetric fates of duplicated genes. BMC Biol..

[18] Kang YJ (2017). CPC2: a fast and accurate coding potential calculator based on sequence intrinsic features. Nucleic Acids Res..

[19] GC Content Calculator—Online Analysis and Plot Tool—BiologicsCorp. Accessed 2023. https://www.biologicscorp.com/tools/GCContent/#.ZFueB3ZBw2x.

[20] Frazer KA, Pachter L, Poliakov A, Rubin EM, Dubchak I (2004). VISTA: computational tools for comparative genomics. Nucleic Acids Res..

[21] Rice, P., Longden, L. & Bleasby, A. EMBOSS: the European molecular biology open software suite. *Trends Genet.* **16**, 10.1016/S0168-9525(00)02024-2 (2000). 10.1016/s0168-9525(00)02024-2 10827456

[22] Luo S (2016). Divergent lncRNAs regulate gene expression and lineage differentiation in pluripotent cells. Cell Stem Cell.

[23] Quinn JJ (2016). Rapid evolutionary turnover underlies conserved lncRNA–genome interactions. Genes Dev..

[24] Lovell JT (2022). GENESPACE tracks regions of interest and gene copy number variation across multiple genomes. eLife.

[25] Emms DM, Kelly S (2019). OrthoFinder: phylogenetic orthology inference for comparative genomics. Genome Biol..

[26] Wang Y (2012). MCScanX: a toolkit for detection and evolutionary analysis of gene synteny and collinearity. Nucleic Acids Res..

[27] Grabherr MG (2010). Genome-wide synteny through highly sensitive sequence alignment: Satsuma. Bioinformatics.

[28] Ross CJ (2021). Uncovering deeply conserved motif combinations in rapidly evolving noncoding sequences. Genome Biol..

[29] Warde-Farley D (2010). The GeneMANIA prediction server: biological network integration for gene prioritization and predicting gene function. Nucleic Acids Res..

[30] Shechner DM, Hacisuleyman E, Younger ST, Rinn JL (2015). Multiplexable, locus-specific targeting of long RNAs with CRISPR-Display. Nat. Methods.

[31] Agarwala S, Sanders TA, Ragsdale CW (2001). Sonic hedgehog control of size and shape in midbrain pattern formation. Science.

[32] Fogel JL, Chiang C, Huang X, Agarwala S (2008). Ventral specification and perturbed boundary formation in the mouse midbrain in the absence of hedgehog signaling. Dev. Dyn..

[33] Statello L, Guo CJ, Chen LL, Huarte M (2021). Gene regulation by long non-coding RNAs and its biological functions. Nat. Rev. Mol. Cell Biol..

[34] Constanty F, Shkumatava A (2021). lncRNAs in development and differentiation: from sequence motifs to functional characterization. Development.

[35] Mattick JS (2023). Long non-coding RNAs: definitions, functions, challenges and recommendations. Nat. Rev. Mol. Cell Biol..

[36] Wang, J. et al. Neutral evolution of ‘non-coding’ complementary DNAs. *Nature* **431**, 1–2 (2004). 15495343

[37] Zhou Y (2010). Activation of paternally expressed genes and perinatal death caused by deletion of the Gtl2 gene. Development.

[38] Stadtfeld M (2010). Aberrant silencing of imprinted genes on chromosome 12qF1 in mouse induced pluripotent stem cells. Nature.

[39] Bell CC (2016). The Evx1/Evx1as gene locus regulates anterior-posterior patterning during gastrulation. Sci. Rep..

[40] Hezroni H (2015). Principles of long noncoding RNA evolution derived from direct comparison of transcriptomes in 17 species. Cell Rep..

[41] Pegueroles C (2019). Transcriptomic analyses reveal groups of co-expressed, syntenic lncRNAs in four species of the genus Caenorhabditis. RNA Biol..

[42] Cabili MN (2015). Localization and abundance analysis of human lncRNAs at single-cell and single-molecule resolution. Genome Biol..

[43] González-Moro I (2023). A long non-coding RNA that harbors a SNP associated with type 2 diabetes regulates the expression of TGM2 gene in pancreatic beta cells. Front. Endocrinol..

[44] Yamaguchi K (2021). Technical considerations in Hi-C scaffolding and evaluation of chromosome-scale genome assemblies. Mol. Ecol..

[45] Altschul, S. F., Gish, W., Miller, W., Myers, E. W. & Lipman, D. J. Basic local alignment search tool. *J. Mol. Biol*. **215**, 403–410 (1990). 10.1016/S0022-2836(05)80360-2 2231712

[46] Camacho C (2009). BLAST+: architecture and applications. BMC Bioinforma..

[47] Robinson, J. T. et al. Integrative genomics viewer. *Nat. Biotechnol.* **29**, 10.1038/nbt.1754 (2011). 10.1038/nbt.1754 PMC3346182 21221095

[48] Cantalapiedra CP, Hern̗andez-Plaza A, Letunic I, Bork P, Huerta-Cepas J (2021). eggNOG-mapper v2: functional annotation, orthology assignments, and domain prediction at the metagenomic scale. Mol. Biol. Evol..

[49] Bailey TL, Johnson J, Grant CE, Noble WS (2015). The MEME suite. Nucleic Acids Res..

[50] Castellanos-Rubio A (2016). A long noncoding RNA associated with susceptibility to celiac disease. Science.

[51] García-Moreno F, Vasistha NA, Begbie J, Molnár Z (2014). CLoNe is a new method to target single progenitors and study their progeny in mouse and chick. Development.

[52] Rueda-Alanã, E. & Garciá-Moreno, F. Time in neurogenesis: conservation of the developmental formation of the cerebellar circuitry. *Brain Behav. Evol.*10.1159/000519068 (2021). 10.1159/000519068 34592741

