# Supplementary material for: Functional evolutionary convergence of long noncoding RNAs involved in embryonic development
Source: Commun Biol. 2023 Sep 5;6:908. doi: 10.1038/s42003-023-05278-z (PMC10480150; doi:10.1038/s42003-023-05278-z)
Supplement: Supplementary file 1 — Supplementary Information [file 42003_2023_5278_MOESM1_ESM.docx]

**SUPPLEMENTARY MATERIALS**

**Supplementary Figures**

**
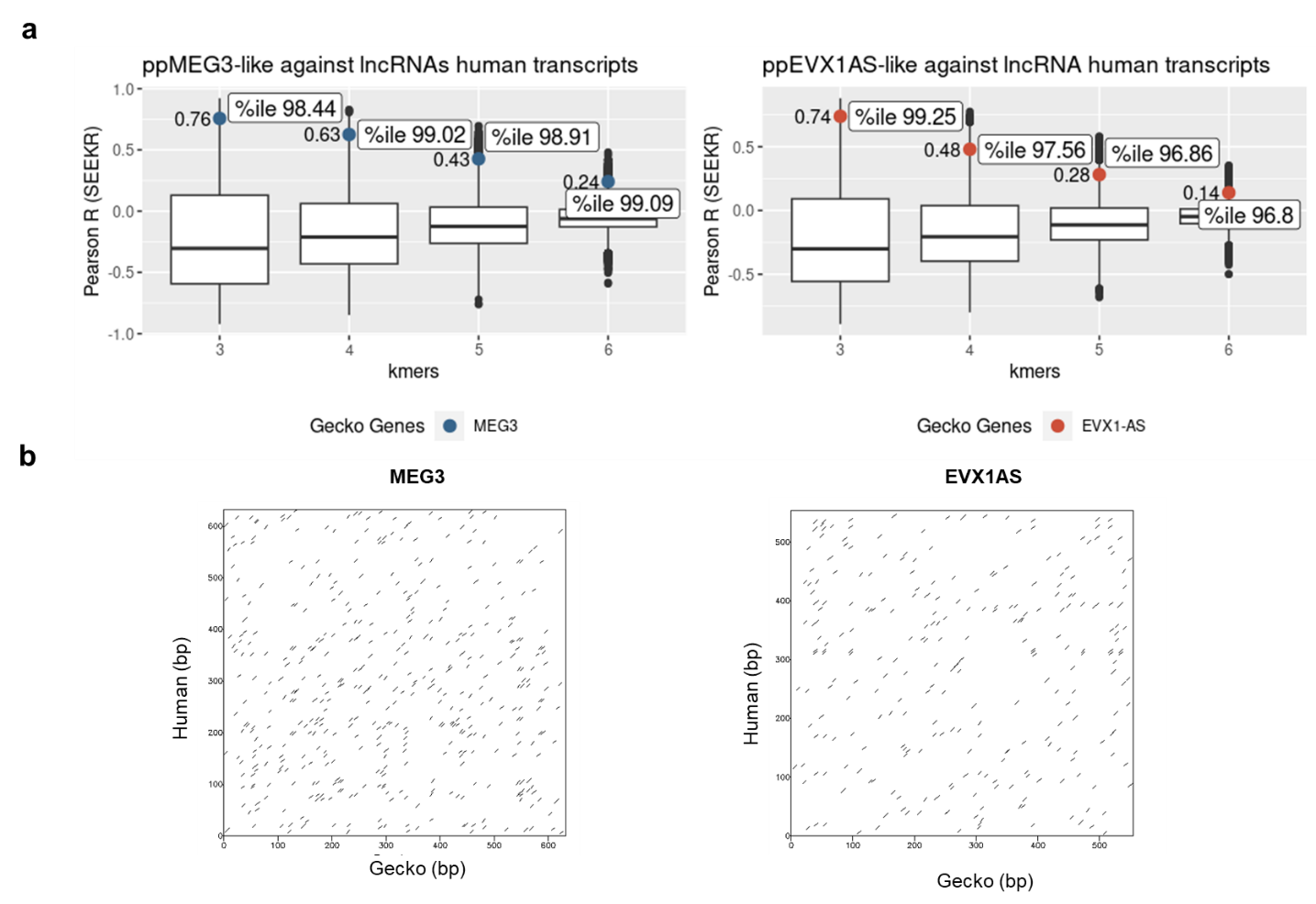
**

**Supplementary Figure 1: (a)** k=3 to k=6 k-mer content analysis of *Pp-MEG3-like* and *Pp-EVX1AS-like* candidates. Candidate lncRNAs were compared against the human lncRNA repository (Gencode v1). Blue and red dots correspond to the *Pp-MEG3-like* and *Pp-EVX1AS-like* genes respectively**.** **(b)** Linear similarity of the human and gecko *MEG3* and *EVX1As* lncRNAs by Dot Plot analysis.


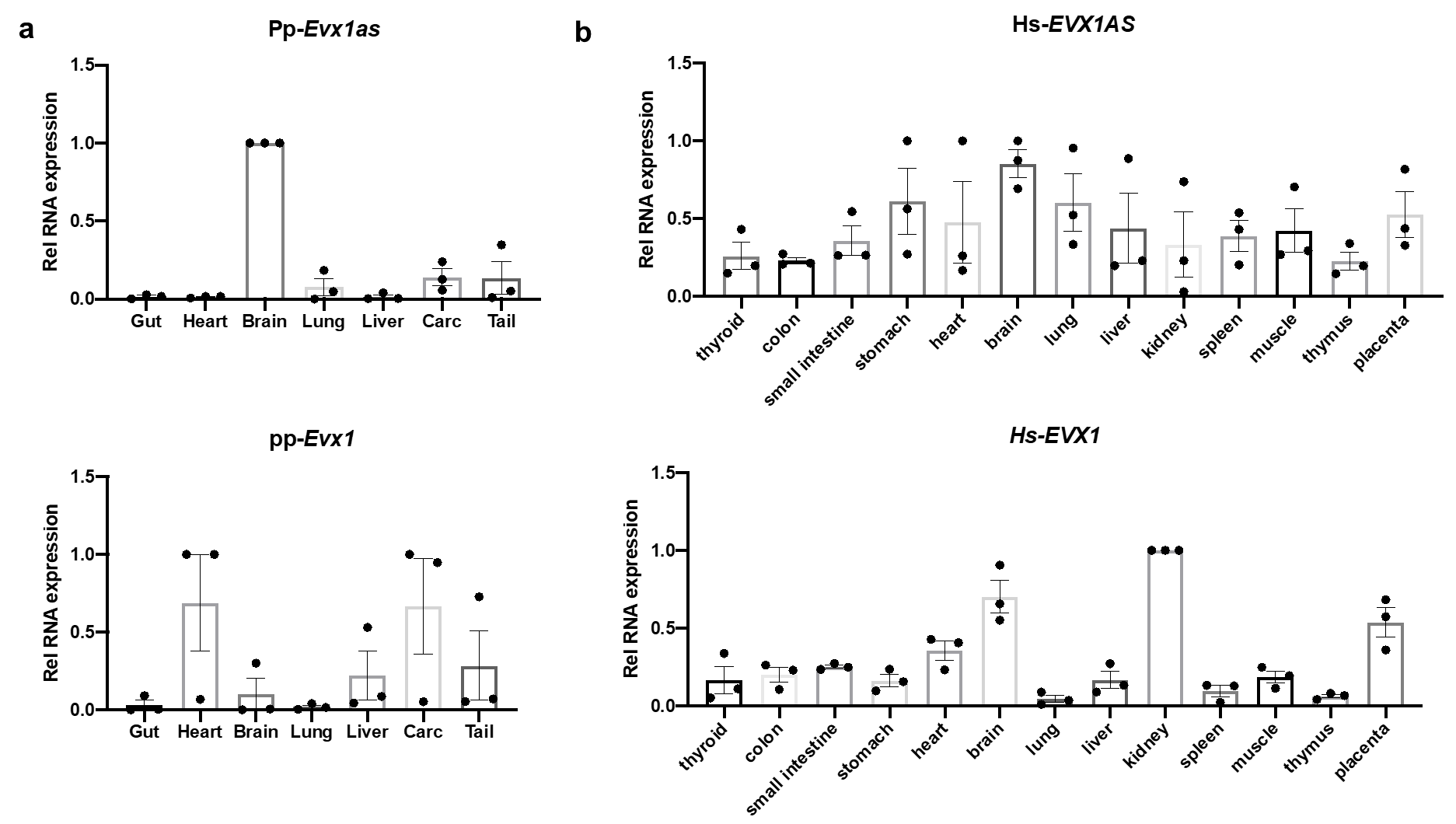


**Supplementary Figure 2:** Relative expression of **(a)** gecko *EVX1AS-like* and *Evx1* and **(b)** human *EVX1AS* and *EVX1* in a range of tissues**.** Gecko and human *RPLP0* were used as housekeeping control. Data represents the mean and standard error of three independent experiments.


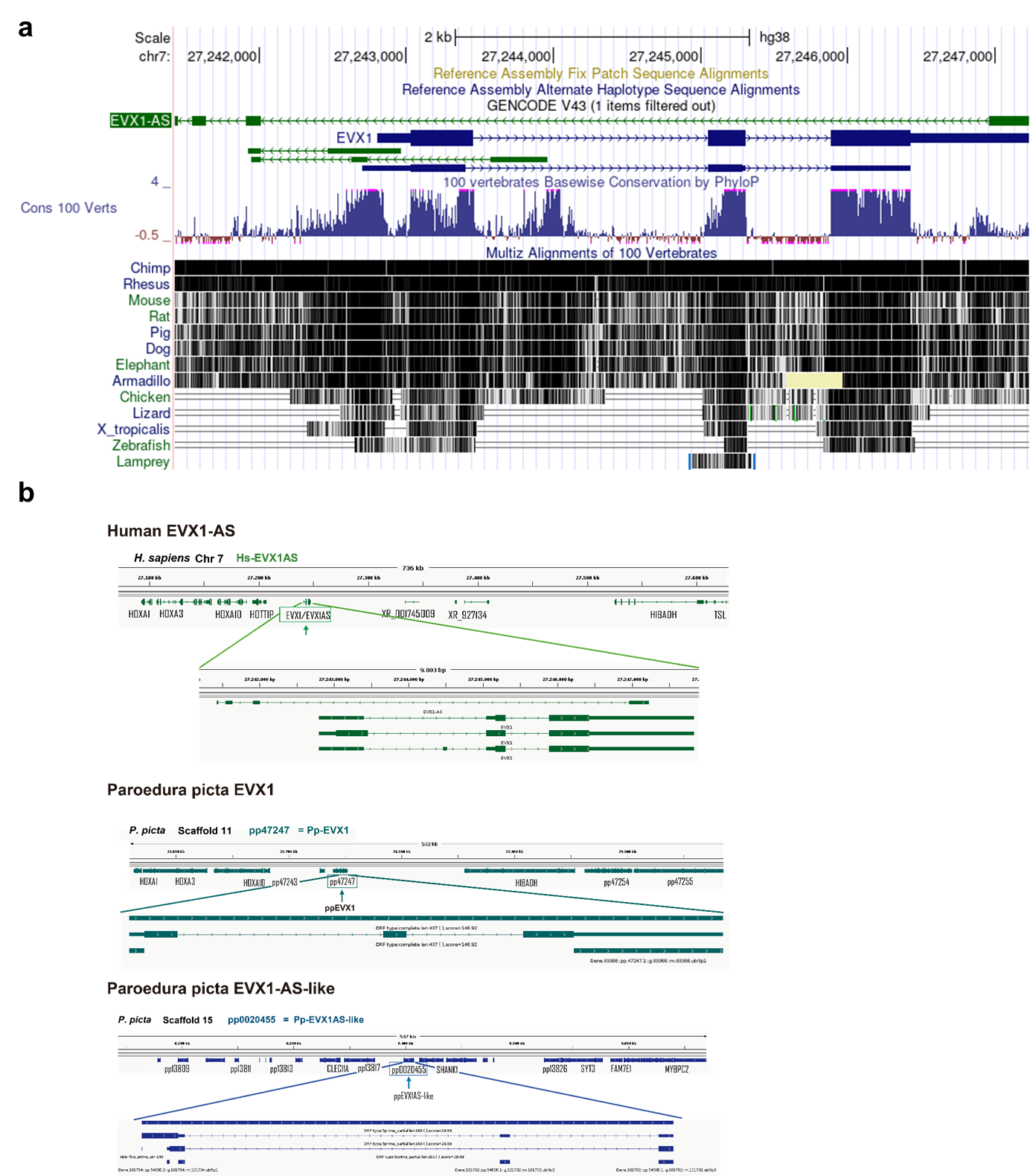


**Supplementary Figure 3: (a)** Evolutionary reconstruction of the *EVX1-EVX1AS* locus using UCSC genome browser. **(b)** Top: Zoomed Genomic locus for *EVX1-AS* human gene (green). Bottom: Zoomed Locus of its functionally equivalent gecko’s genes, pp0020455 ≈ *pp-EVX1AS-like* (blue). Genome visualization is carried out by Integrative Genome Viewer (IGV) with Human (GRCh38/hg38, RefSeq.gtf) and *P. picta* (v2, BRAKER.gtf) genomes, respectively. Except for pp0020455, which retains the V1 annotation.

**
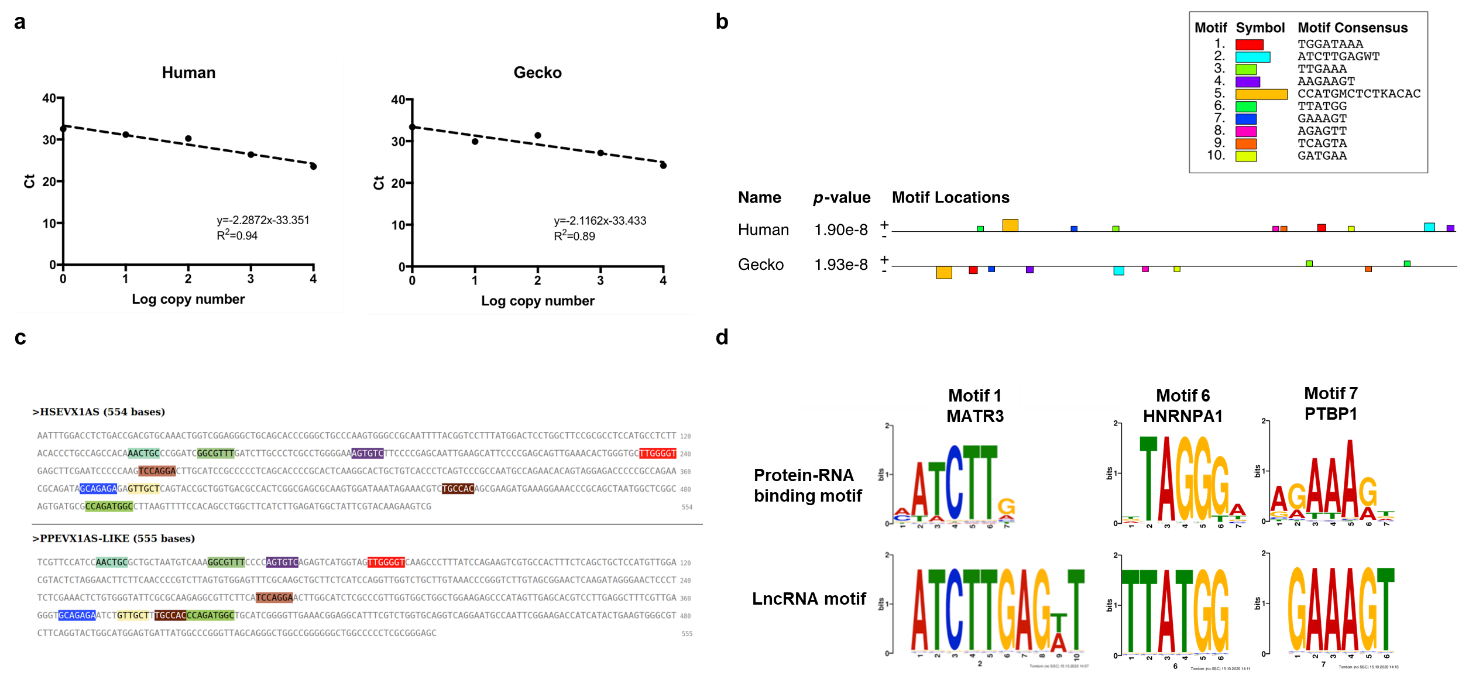
**

**Supplementary Figure 4: (a)** Standard curve for the human (top) and gecko (bottom) *EVX1AS* copy number analyses. **(b)** Common enriched motifs in human and gecko *EVX1AS* lncRNA using MEME Motif Discovery tool. **(c)** Conserved motifs as described by lncLOOMv2 analysis. **(D)** Alignment of the enriched lncRNA motifs with described RNA binding protein motifs using TOMTOM Motif comparison tool.

**Supplementary Figure 5: (a)** Schematic representation of the RNA tethering experiments. **(b)** Schematic representation of the *in vitro* (left) and *in vivo* (right) experiments. Relative expression of *GAPDH* negative control in SHSY5Y cells after *Hs-EVX1AS* **(c)** and *Pp-EVX1AS-like* **(d)** overexpression. **(e)** Relative RNA expression of human lncRNAs (*EVX1AS* and negative control lncRNA) and human *EVX1* upon *EVX1AS* or negative control lncRNA overexpression in SHSY5Y cells. **(f)** Relative expression of *GAPDH* negative control in SHSY5Y cells after human *EVX1AS* and negative control lncRNA overexpression. *RPLP0* was used as housekeeping control and values were normalized to the highest values in each experiment. **(g)** *Evx1* expression analysis in chicken embryonic mesencephalon (Ms) and forebrain (Fb). Data represents the mean and standard error of brain tissue from 3 chicken embryos. **(h)** Immunohistochemistry of the chicken embryonic midbrain on coronal section showing the successful transfection of the control plasmid. Transfected cells are shown in red due to their ectopic expression of red fluorescent protein RFP. LHX5 and MEIS2 patterns of expression (in green) indicate the mesencephalic region that was actually transfected. PH3 immunostaining shows mitotic cells at the germinative zone of the mesencephalon, in white. DAPI counterstain in blue. Relative expression of *Gapdh* negative control in chicken mesencephalon after *Hs-EVX1AS.* Scale bars reprensent 1mm (right) and 100micras (left) **(i)** and *Pp-EVX1AS-like* **(j)** overexpression. Data represents the mean and standard error of at least three independent experiments. HS1, HS2 and GG1, GG2 correspond to the two different sgRNAs used for human and chicken transfections respectively. (*p<0.05, **p<0.01, ****p<0.0001 according to one tailed Student’s t-test).

**Supplementary Tables**

**Supplementary Table 1. List of used primers and sequences**

| **GENE NAME** | **SPECIES** | **Fw primer sequence** | **Rv primer sequence** |
| --- | --- | --- | --- |
| *EVX1AS* | Human | TCTCCTCCAAATGACTGATGGC | AACGGGAAAGCGACTTCTTG |
| *EVX1* | Human | AACCCCAATGCAAGCTTCAC | TGGGCTTCAAAGGAAAACCC |
| *RPLP0* | Human | GCAGCATCTACAACCCTGAAG | CACTGGCAACATTGCGGAC |
| *GAPDH* | Human | AAGGTGAAGGTCGGAGTC | CCCATACGACTGCAAAGACC |
| *pp-EVX1AS-like* | Gecko | TCAACGAAAGCCTCAAGGAC | AGGTGGAATTCGGAACCAGATG |
| *Evx1* | Gecko | TTTGGTGGGTGTGCTTTACC | AGCAACTGACAATGGAAGGC |
| *pp-MEG3-like* | Gecko | TGCTTTTGTTTGGGGACAGC | AACCACCTCTACAACCACGAAG |
| *Rplp0* | Gecko | AAGCCACCTTGCTGAACATG | ATGCTGCCGTTGTCAAACAC |
| *Evx1* | Chicken | TGCCAGAAACCACCATCAAG | GTGGCTCATCATGTAGGTGTAG |
| *Gapdh* | Chicken | AACATCATCCCAGCGTCCAC | AACAGAGACATTGGGGGTTGG |
| *Rplp7* | Chicken | GAAGCGCCTTGGAAAACTTGG | TGAAGGGCCAAAGGAAGTTGT |
