# Supplementary material for: Functional evolutionary convergence of long noncoding RNAs involved in embryonic development
Source: Commun Biol. 2023 Sep 5;6:908. doi: 10.1038/s42003-023-05278-z (PMC10480150; doi:10.1038/s42003-023-05278-z)
Supplement: Supplementary file 2 — Description of Additional Supplementary Files [file 42003_2023_5278_MOESM2_ESM.pdf]

Description of Additional Supplementary Files

File name: Supplementary Data 1

Description: Numerical source data for graphs and charts.
