# Supplementary material for: Functional evolutionary convergence of long noncoding RNAs involved in embryonic development
Source: Commun Biol. 2023 Sep 5;6:908. doi: 10.1038/s42003-023-05278-z (PMC10480150; doi:10.1038/s42003-023-05278-z)
Supplement: Supplementary file 4 — Reporting Summary [file 42003_2023_5278_MOESM4_ESM.pdf]

nature portfolio

Corresponding author(s): *Double-blind peer review submissions: write DBPR and your manuscript number here instead of author names.*

Last updated by author(s): Aug 11, 2023

Reporting Summary

Nature Portfolio wishes to improve the reproducibility of the work that we publish. This form provides structure for consistency and transparency in reporting. For further information on Nature Portfolio policies, see our Editorial Policies and the Editorial Policy Checklist.

Statistics

For all statistical analyses, confirm that the following items are present in the figure legend, table legend, main text, or Methods section.

n/a	Confirmed	
☐	☒	The exact sample size (*n*) for each experimental group/condition, given as a discrete number and unit of measurement
☐	☒	A statement on whether measurements were taken from distinct samples or whether the same sample was measured repeatedly
☐	☒	The statistical test(s) used AND whether they are one- or two-sided<br>*Only common tests should be described solely by name; describe more complex techniques in the Methods section.*
☒	☐	A description of all covariates tested
☐	☒	A description of any assumptions or corrections, such as tests of normality and adjustment for multiple comparisons
☐	☒	A full description of the statistical parameters including central tendency (e.g. means) or other basic estimates (e.g. regression coefficient) AND variation (e.g. standard deviation) or associated estimates of uncertainty (e.g. confidence intervals)
☐	☒	For null hypothesis testing, the test statistic (e.g. *F*, *t*, *r*) with confidence intervals, effect sizes, degrees of freedom and *P* value noted<br>*Give P values as exact values whenever suitable.*
☒	☐	For Bayesian analysis, information on the choice of priors and Markov chain Monte Carlo settings
☒	☐	For hierarchical and complex designs, identification of the appropriate level for tests and full reporting of outcomes
☒	☐	Estimates of effect sizes (e.g. Cohen's *d*, Pearson's *r*), indicating how they were calculated

Our web collection on statistics for biologists contains articles on many of the points above.

Software and code

Policy information about availability of computer code

Data collection	Genomic resources were downloaded from the Paroedura picta repository (https://transcriptome.riken.jp/reptiliomix/). Transcriptome (Hara et al., 2015) and genome (Paroedura picta v1) were used for this analysis, as P.picta genome v2 resources was not available at the accession time (February 2020). Likewise, the sequences for the human transcripts for EVX1AS, MEG3, NEAT1 were downloaded from UCSC. The used sequences are available at our github repository (https://github.com/rodrisenovilla/Olazagoitia-Garmendia).
Data analysis	A Jupyter Notebook has been uploaded on GitHub to follow all the steps for k-mer and CROSSalign analysis in a Python3 environment: https://github.com/rodrisenovilla/Olazagoitia-Garmendia/blob/ffbeb12b889791b4b0c24f47cff4436cce2979eb/seekr.ipynb<br>Although many of the analysis were carried out in web-page apps, we have coded a pipeline to unify all our bioinformatic analysis (Olazagoitia_pipeline.ipynb), to generate the boxplots for SEEKR results (seekr_output_explore.Rmd) and to carry out GENESPACE pipeline (Genespace_pipeline.Rmd). The files used for the analysis can be found in our GitHub (https://github.com/rodrisenovilla/Olazagoitia-Garmendia).

For manuscripts utilizing custom algorithms or software that are central to the research but not yet described in published literature, software must be made available to editors and reviewers. We strongly encourage code deposition in a community repository (e.g. GitHub). See the Nature Portfolio guidelines for submitting code & software for further information.

Data

Policy information about availability of data

All manuscripts must include a data availability statement. This statement should provide the following information, where applicable:

- Accession codes, unique identifiers, or web links for publicly available datasets
- A description of any restrictions on data availability
- For clinical datasets or third party data, please ensure that the statement adheres to our policy

All code data is available in Github (https://github.com/rodrisenovilla/Olazagoitia-Garmendia)

Human research participants

Policy information about studies involving human research participants and Sex and Gender in Research.

Reporting on sex and gender	*Use the terms sex (biological attribute) and gender (shaped by social and cultural circumstances) carefully in order to avoid confusing both terms. Indicate if findings apply to only one sex or gender; describe whether sex and gender were considered in study design whether sex and/or gender was determined based on self-reporting or assigned and methods used. Provide in the source data disaggregated sex and gender data where this information has been collected, and consent has been obtained for sharing of individual-level data; provide overall numbers in this Reporting Summary. Please state if this information has not been collected. Report sex- and gender-based analyses where performed, justify reasons for lack of sex- and gender-based analysis.*
Population characteristics	*Describe the covariate-relevant population characteristics of the human research participants (e.g. age, genotypic information, past and current diagnosis and treatment categories). If you filled out the behavioural & social sciences study design questions and have nothing to add here, write "See above."*
Recruitment	*Describe how participants were recruited. Outline any potential self-selection bias or other biases that may be present and how these are likely to impact results.*
Ethics oversight	*Identify the organization(s) that approved the study protocol.*

Note that full information on the approval of the study protocol must also be provided in the manuscript.

Field-specific reporting

Please select the one below that is the best fit for your research. If you are not sure, read the appropriate sections before making your selection.

☒ Life sciences ☐ Behavioural & social sciences ☐ Ecological, evolutionary & environmental sciences

For a reference copy of the document with all sections, see nature.com/documents/nr-reporting-summary-flat.pdf

Life sciences study design

All studies must disclose on these points even when the disclosure is negative.

Sample size	In vitro and in ovo experiments were performed at least in 3 replicates
Data exclusions	Data were not excluded for the analyses
Replication	All experimental findings were replicated
Randomization	Samples were not randomized as groups were separated depending on the plasmid used in each case, i.e. empty vector pCMV6, hs-EVX1AS overexpressing plasmid or pp-EVX1AS like overexpressing plasmid.
Blinding	Blinding was not possible during these experiments, as relative expression analyses comparing to control groups have been performed.

Reporting for specific materials, systems and methods

We require information from authors about some types of materials, experimental systems and methods used in many studies. Here, indicate whether each material, system or method listed is relevant to your study. If you are not sure if a list item applies to your research, read the appropriate section before selecting a response.

Materials & experimental systems

n/a	Involved in the study
☐	☒ Antibodies
☐	☒ Eukaryotic cell lines
☒	☐ Palaeontology and archaeology
☐	☒ Animals and other organisms
☒	☐ Clinical data
☒	☐ Dual use research of concern

Methods

n/a	Involved in the study
☒	☐ ChIP-seq
☒	☐ Flow cytometry
☒	☐ MRI-based neuroimaging

Antibodies

Antibodies used

- Rabbit antibody to histone H3 - phospho S10 (Abcam; ab47297), polyclonal, IgG
- Mouse antibody to LHX5 (DSHB; PRCP-LHX5-1B7); monoclonal, clone PCRP-LHX5-1B7.
- Mouse antibody to Meis2 (DSHB; PCRP-MEIS2-1A11); monoclonal, clone PCRP-MEIS2-1A11.
- Goat anti-Rabbit IgG (H+L) Highly Cross-Adsorbed Secondary Antibody, Alexa Fluor™ Plus 647 (Molecular Probes, A32733); polyclonal, IgG
- Goat anti-Mouse IgG (H+L) Cross-Adsorbed Secondary Antibody, Alexa Fluor™ 488 (Molecular Probes, A11001); polyclonal, IgG
- Mouse antibody to MED1 (Santa Cruz Biotechnology; sc-74475), monoclonal, IgG.
- Rabbit antibody to HSP90 (Cell Signaling; #4874) , polyclonal, IgG.
- Rabbit Anti-Mouse IgG (Light Chain Specific) (D3V2A) mAb (HRP Conjugate) (Cell Signalling, #58802)
- Mouse monoclonal [SB62a] Anti-Rabbit IgG light chain (HRP) (Abcam, ab99697)

Validation

- Rabbit antibody to histone H3 - phospho S10, validation and relevant publications as in https://www.abcam.com/histone-h3-phospho-s10-antibody-ab47297.html?productWallTab=ShowAll
- Mouse antibody to LHX5, this antibody has been characterized by the NIH Protein Capture Reagents Program. RRID:AB_2722232. Validation and relevant publications in https://dshb.biology.uiowa.edu/PCRP-LHX5-1B7
- Mouse antibody to Meis2, this antibody has been characterized by the NIH Protein Capture Reagents Program. RRID: AB_2618842. Validation and relevant publications in https://dshb.biology.uiowa.edu/PCRP-MEIS2-1A11
- Goat anti-Rabbit IgG (H+L) Highly Cross-Adsorbed Secondary Antibody, Alexa Fluor™ Plus 647 Validation and relevant publications in https://www.thermofisher.com/antibody/product/Goat-anti-Rabbit-IgG-H-L-Highly-Cross-Adsorbed-Secondary-Antibody-Polyclonal/A32733
- Goat anti-Mouse IgG (H+L) Cross-Adsorbed Secondary Antibody, Alexa Fluor™ 488. Validation and references in https://www.thermofisher.com/antibody/product/Goat-anti-Mouse-IgG-H-L-Cross-Adsorbed-Secondary-Antibody-Polyclonal/A-11001
- Mouse antibody to MED1 Validation and references in https://www.scbt.com/p/trap220-antibody-h-7
- Mouse antibody to GAPDH Validation and references in https://www.scbt.com/p/gapdh-antibody-0411?requestFrom=search
- Rabbit Anti-Mouse IgG (Light Chain Specific) (D3V2A) mAb (HRP Conjugate) Validation and references in https://www.cellsignal.com/products/secondary-antibodies/rabbit-anti-mouse-igg-light-chain-specific-d3v2a-mab-hrp-conjugate/58802
- Mouse monoclonal [SB62a] Anti-Rabbit IgG light chain (HRP) (Abcam, ab99697) Validation and relevant publications in https://www.abcam.com/products/secondary-antibodies/mouse-monoclonal-sb62a-rabbit-igg-light-chain-hrp-ab99697.html

Eukaryotic cell lines

Policy information about cell lines and Sex and Gender in Research

Cell line source(s)	SHSY5Y cells were purchased from ATCC
Authentication	Cell lines were not authenticated
Mycoplasma contamination	Cells were tested for mycoplasma every month
Commonly misidentified lines (See ICLAC register)	*Name any commonly misidentified cell lines used in the study and provide a rationale for their use.*

Animals and other research organisms

Policy information about studies involving animals; ARRIVE guidelines recommended for reporting animal research, and Sex and Gender in Research

Laboratory animals	As experimental animals, we used chick embryos from E3 to E6
Wild animals	N/A
Reporting on sex	N/A
Field-collected samples	N/A

Ethics oversight | Chick embryos are not protected by the current European (2010/63/EU) and Spanish (RD 53/2013) regulations, so no specific licenses or approval of the bioethical commitee were required.

Note that full information on the approval of the study protocol must also be provided in the manuscript.
